# Unraveling the Different Drivers of PM_2.5_ Mass and Oxidative Potential at Two Sites of Southern Italy

**DOI:** 10.1021/acs.est.6c02676

**Published:** 2026-05-25

**Authors:** Serena Potì, Laura Martina, Florin Unga, Daniela Cesari, Adelaide Dinoi, Antonio Pennetta, Ermelinda Bloise, Paola Semeraro, Giuseppe Deluca, Luca Cirillo Ciricugno, Livia Giotta, Maria Giulia Lionetto, Lucio Calcagnile, Annarosa Mangone, Maria Rachele Guascito, Daniele Contini

**Affiliations:** 1 Istituto di Scienze dell’Atmosfera e del Clima (ISAC), Consiglio Nazionale delle Ricerche (CNR), Str. Prv. Lecce-Monteroni km 1.2, Lecce 73100, Italy; 2 Dipartimento di Ingegneria dell’Innovazione, Università del Salento, Via Per Monteroni 165, Lecce 73100, Italy; 3 Dipartimento di Scienze e Tecnologie Biologiche ed Ambientali DiSTeBA, Università del Salento, Via Per Monteroni 165, Lecce 73100, Italy; 4 Dipartimento di Matematica e Fisica, Università del Salento, Via Per Arnesano, Lecce 73100, Italy; 5 Dipartimento di Chimica, Università degli Studi di Bari, Via Orabona 4, Bari 70126, Italy

**Keywords:** source apportionment, oxidative potential, acellular assays, DTT, AA, PM_2.5_ solubility

## Abstract

The oxidative potential (OP) of PM_2.5_ was investigated during two measurement campaigns in 2024 (winter and summer) done simultaneously at an urban background and a traffic site. The research provides unprecedented chemical detail for this region, integrating elemental analysis (ED-XRF), carbonaceous fractions (OC/EC, WSOC), major ions, organic tracers (sugars/levoglucosan), and water-soluble organic nitrogen (WSON). The OP was quantified by using two assays: dithiothreitol (OP^DTT^) and ascorbic acid (OP^AA^). Source apportionment revealed competing trends of sources limiting spatial and seasonal variabilities and distinct drivers for the two OP assays. Traffic emerged as the primary contributor to OP^AA^ at both sites, while OP^DTT^ was influenced by traffic, secondary organic aerosols (SOAs), biomass burning, and resuspension/construction. Sea spray, nitrate, and construction-related emissions significantly impacted OP^DTT^ but had a negligible effect on OP^AA^. Primary biological aerosols (fungal spores) influenced the OP^AA^. Seasonal variations showed dominance of traffic and biomass burning in winter (50–60% of OP), whereas sulfate and SOA became prominent during summer, for OP^DTT^. OP^AA^ peaked in summer, while OP^DTT^ peaked in winter. Traffic-induced SOA exhibits a higher redox activity relative to its mass contribution, opposite to sulfate. Season-dependent mitigation strategies could be useful to effectively reduce the oxidative burden of PM_2.5_.

## Graphical abstract

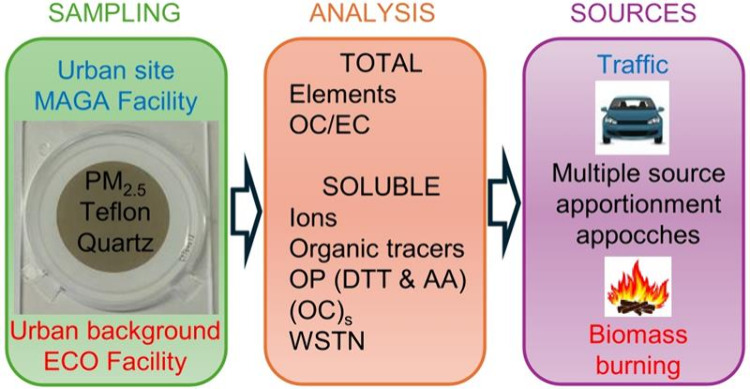

## 1 Introduction

Atmospheric particulate matter (PM) poses a significant threat to human health, constituting an economic and societal challenge for policymakers,^1^ and it is nowadays the largest environmental risk to global health.^2^ There is a growing consensus that the harmful effects of PM may be mainly related to mechanisms of oxidative stress.^3–5^ Oxidative stress is the imbalance between oxidizing and antioxidant species at the cellular level.^6^ The oxidative potential (OP) of particulate matter, defined as the ability of PM to transport reactive oxygen species (ROS) or to catalyze their formation when inhaled, has emerged in recent years as a potential global indicator of health-related effects.^3,7^ The new EU Ambient Air Quality Directive (2024/2881) includes strict target values for PM_2.5_ concentrations and gives to OP policy relevance being now included as a pollutant of emerging concern to be monitored, calling for more epidemiological evidence to guide future regulation. A standardized assay and protocol for determination of OP is not yet available,^8^ even though the most widely used approaches are based on the DTT (dithiothreitol) assay and on the ascorbic acid (AA) assay.^9^ In this framework, a relevant aspect of current research is the evaluation of the natural and anthropogenic sources driving OP rather than PM_2.5_ concentrations.^10–12^

To the best of our knowledge, there are no source apportionment studies of oxidative potential of PM_2.5_ at multiple sites focusing on both assays in Italy. However, there are studies at multiple sites focusing on DTT assay of PM_2.5_ in south Italy^13,14^ or studies addressing both assays for PM_10_ in central Italy.^15^ Other studies compared the source apportionment of OP in PM_2.5_ with multiple assays at different sites in Europe,^16^ East Mediterranean,^17^ USA,^11,18^ and China.^19^ The results consistently show that the main sources driving OP^DTT^ and OP^AA^ are different, with large variability in the role of sources such as secondary aerosol, aged aerosol, and crustal material. The formation of secondary organic aerosols (SOA) may significantly influence the OP of particulate matter, making aged particles even more hazardous than primary precursors.^10^ However, source apportionment based on traditional data sets struggles to distinguish between secondary organic and inorganic aerosols, leading to uncertainties on source apportionment of sources such as secondary sulfate^14^ or secondary nitrate.^20^

Another relevant aspect is that source apportionment of OP is frequently conducted by deriving source-specific intrinsic (i.e., mass-normalized) values. However, these values often exhibit significant interstudy variability, which limits comparability due to several factors. These include storage artifacts involving short-lived reactive components^21^ and discrepancies in OP measurements (even within the same assay) arising from variations in analytical protocols and extraction procedures.^8^ Furthermore, actual differences in the chemical profiles of local source, such as those induced by vehicle fleet composition or the type of biomass burned for heating, along with uncertainties inherent to different source apportionment models,^20^ contribute to this variability. Additionally, mass size distributions can influence the comparability of results, as specific sources drive the OP of particles across different size ranges.^22–24^

This study aims to expand current knowledge on the oxidative potential by addressing some of the key research gaps. Specifically, the sources driving OP^AA^ and OP^DTT^ of PM_2.5_ were investigated at two sites in southern Italy using an unprecedentedly detailed chemical characterization for this region. This approach enabled the differentiation between SOA and secondary inorganic species (mainly sulfate and nitrate salts), local soil and long-range transport of dust, and fresh versus aged marine aerosols. By comparing three source apportionment methods, the research explored the competing seasonal and spatial trends that influence OP drivers in the region and limits the overall cumulative variability. Moreover, the findings are indicative and promising for developing season-dependent mitigation strategies to effectively reduce the oxidative burden of atmospheric fine particles.

## 2 Material and Methods

### 2.1 Measurement Sites, Instruments, and Sampling Strategy

Two measurement campaigns were done simultaneously at two sites: an urban background site and an urban site in the area of Lecce in southern Italy (Figure S2). The urban background site was the Environmental-Climate Observatory (ECO, 40°20′8″ N-18°07′28″ E) of Lecce, regional station of the Global Atmosphere Watch (GAW-WMO program) and part of the ACTRIS network. The urban site was located at approximately 4.3 km northeast of ECO, in the center of the town of Lecce (95 700 inhabitants in the Salento peninsula), facing a four-lane road with traffic volumes reaching up to 2400 vehicles per hour.^25^ This site was investigated using the mobile laboratory for aerosol and gas measurements (MAGA, 40°35′6″N-18°16′7″E) that is an exploratory platform of the ACTRIS network. This area of south Italy is mainly influenced by local anthropogenic sources, secondary and photochemical pollution, sporadic medium range transport of pollution from industrial settlements located between 45 and 80 km in the NNW-NW direction, and long-range transport of Saharan dust.^14^ In addition, the area is frequently affected by new particle formation (NPF) events (i.e., nucleation), which occur on roughly 25% of days.^26^

Two measurements campaigns were performed simultaneously at the two sites: one in the winter to early spring period (February–April 2024) and the other in the summer period (June–August 2024). Daily (starting at midnight) PM_2.5_ samples were simultaneously collected on quartz fiber filters (Whatman, 47 mm), thermally treated (700 °C for 2 h) to limit their initial contamination, and on Teflon (Whatman, 47 mm with ring, porosity 2 μm) filters using two low-volume (2.3 m^3^/h) dual channel samplers (FAI Instruments S.r.l., Italy). In total, 300 valid samples for each substrate were collected, uniformly distributed among the two sites. PM_2.5_ concentration on quartz filters was determined automatically by the β-attenuation method while the concentration on Teflon substrates was determined using the reference gravimetric method (UNI EN 12341:2014). Teflon filters were conditioned (for 48h) in a climatised room and weighted with a microbalance (Sartorious Cubis, model MSA6.6S, ±1 μg) before and after sampling. Concentrations were obtained by subtracting values found on field blank filters (four for each campaign and each substrate). Uncertainties were calculated combining the standard deviation of blanks and the expected error on sampled volumes^27^ and ranged between 0.3 and 1.2 μg/m^3^.

### 2.2 Determination of Chemical Composition

Nondestructive ED-XRF analysis was performed to determine the concentration of main elements using a Spectro (XEPOS05) instrument on both substrates following the method reported in Unga et al. (2025).^28^ The instrument was calibrated using 23 medium-concentration elemental thin-film standards from Micromatter. The comparison of measurements on the two substrates was used as QA/QC for the measurements; however, data collected on Teflon were used in the following because this substrate allowed us to determine the concentration of a large set of elements with a lower LOD.^28^ The quantified elements were Na, Mg, Al, Si, P, Cl, K, Ca, Ti, Cr, Mn, Fe, Ni, Cu, Zn, Br, Rb, Sr, and Pb.

Successively, each quartz filter was cut into four quarters. One quarter was used to obtain a punch (1 cm^2^) to measure the concentrations of organic carbon (OC) and elemental carbon (EC), using the thermo-optical analysis (Sunset OC/EC analyzer). This was operated with the EUSAAR2 protocol,^29^ and a sucrose standard solution (2.198 g/L in water, CPAchem Ltd.) was used for the multipoint calibration of the instrument.^30^

The second quarter of the filter was extracted in 12 mL of Milli-Q water (18 MΩ, Carlo Erba) with sonication for 30 min below 30 °C. Extracts were used for the determination of the water-soluble total carbon (WSTC) and total nitrogen (WSTN) using a TOC/TN total organic carbon analyzer (Shimadzu, model TOC-LCPH/CPN). The approach used was based on the work of Pennetta et al. (2026).^31^ The sample aliquot was injected into a chamber where thermo-oxidative combustion at 720 °C converts carbon and nitrogen into carbon dioxide and nitric oxide, respectively. While CO_2_ is detected by a nondispersive infrared (NDIR) analyzer, NO reacts with ozone and produces electromagnetic radiation that is detected by a chemiluminescence detector. To quantify the WSTC and WSTN, multipoint calibration curves of standard solutions of potassium phthalate and potassium nitrate were used, respectively. Measurements indicated that the content of water-soluble inorganic carbon (WSIC) was negligible with values comparable to blanks as happened also in a previous study.^14^ Therefore, the WSTC was essentially comparable with the organic water-soluble fraction of carbon.

Another quarter of each filter was extracted in 3 mL of ultrapure water in an ultrasonic bath for 30 min. The extracts were filtered through a 0.45 μm PTFE membrane and used for determination of major ions and sugars. Cations (Na^+^, NH_4_^+^, K^+^, Mg^2+^, Ca^2+^) were determined using a Dionex Integrion HPIC System (Thermo Fisher Scientific Inc., Waltham, MA, USA) equipped with a Dionex IonPac CG16-4 μm guard column (2 mm × 50 mm) and a CS16-4 μm analytical column (2 mm × 250 mm). The eluent, 30 mM methanesulfonic acid (MSA), was generated by a Dionex EGC 500 MSA cartridge with CR-CTC 600 trap column. Detection was performed by suppressed conductivity using a Dionex CDRS 600 suppressor (2 mm, recycle mode) operating at 15 mA in constant current mode. The system ran in isocratic mode at a flow rate of 0.16 mL min^–1^, with an injection volume of 25 μL and a column temperature of 40 °C. The total run time was 30 min.

Anions (Cl^–^, NO_2_^–^, Br^–^, NO_3_^–^, C_4_H_4_O_4_^2–^, SO_4_^2–^, C_2_O_4_^2–^) and sugars (levoglucosan, mannitol, mannosan, galactosan, glucose, mannose) were analyzed using a Dionex ICS-6000 HPIC dual-channel system (Thermo Fisher Scientific Inc.). One channel was dedicated to anions and the other to sugars. For anions, separation was achieved with a Dionex IonPac AG11-HC-4 μm guard column (2 mm × 50 mm) and an AS11-HC-4 μm analytical column (2 mm × 250 mm). The eluent was KOH, applied as a gradient from 1 mM (0–5 min) to 30 mM (5–42 min), then back to 1 mM (42–43 min), at a flow rate of 0.38 mL min^–1^. Suppression was performed using a Dionex ERS 500e electrolytically regenerated suppressor (2 mm) at 29 mA. The total run time was 47 min. For sugars, the same ICS-6000 system was equipped with a Dionex CarboPac PA300-4 μm column set (guard + analytical). The eluent was 15 mM KOH in isocratic mode at a flow rate of 0.25 mL min^–1^. Detection was carried out by integrated amperometry using a quadruple-pulse waveform with an Ag/AgCl reference electrode. The column temperature was maintained at 30 °C, and the run time was 21 min.

The concentration of each chemical species was evaluated with blank subtraction, using eight blanks for each substrate and each campaign. The estimated uncertainties are discussed in the Supporting Information (Table S1).

### 2.3 Determination of Oxidative Potential

A quarter of each filter was extracted in 12 mL of ultrapure Milli-Q water (18 MΩ) and sonicated for 30 min in an ultrasonic bath. These extracts were filtered through 0.45 μm PTFE membrane and used for the OP determination using both DTT and AA assays.

The DTT assay was performed following a protocol adapted from Cho et al. (2005)^32^ and successive updates,^33^ maintaining the original volume ratio, molar ratios, and working DTT concentration of 100 μM (dithiothreitol; CAS 3483-12-3; Sigma-Aldrich). Analyses were done using a FLUOstar Omega UV–Vis microplate reader (BMG Labtech) equipped with two autoinjectors and 48-well Greiner plates, temperature-controlled at 37 °C throughout the measurements. The DTT depletion was monitored over 45 min, a reaction time selected within the range established by Cho et al. (2005),^32^ as reagent depletion at 100 μM was verified to be linear over this time window in the measurements reported in this work. The reaction mixture was prepared by combining 4.5 mL of extract with 0.5 mL of 0.5 M phosphate buffer (PB, pH 7.4). Aliquots of 665 μL were dispensed into wells corresponding to reaction times of 0, 5, 10, 15, 20, and 45 min. Matrix absorbance (Abs_mat_) was measured at 412 nm prior to initiating the reaction. Successively, DTT was injected (35 μL of a 2 mM solution), obtaining an initial concentration of 100 μM (70 nmol per well). At each time-point, the reaction was quenched by automatic addition of 80 μL TCA (trichloroacetic acid, 10% w/v; CAS 76-03-9; Sigma-Aldrich). After 45 min, 420 μL of a DTNB solution prepared in Tris-HCl buffer (0.4 M, pH 8.9) + 20 mM EDTA was added to each well, maintaining a DTNB:DTT molar ratio of 10:1. Residual DTT reacted with DTNB to form TNB, which was quantified by measuring absorbance at 412 nm. Absorbance values were converted to nmol of residual DTT using an external calibration curve. The OP activity was calculated as the slope of the linear regression of nmol of residual DTT versus time.

The AA assay was conducted using the same instrument but using UV-transparent 96-well Greiner plates maintained at 37 °C throughout the measurements. The AA assay was performed following a semiautomated protocol adapted from Calas et al. (2018),^33^ maintaining a working AA concentration of 100 μM (ascorbic acid; CAS 50-81-7; Sigma-Aldrich). For each well, 300 μL of aqueous PM extract was dispensed, and matrix absorbance (Abs_mat_) at 265 nm was recorded prior to AA addition. The reaction was initiated by automatic injection of 33 μL of 1 mM AA solution prepared in 0.5 M PB, resulting in a final concentration of 100 μM (33 nmol AA per well). Absorbance was measured immediately (*t* = 0) and at 5 min intervals for 45 min. Absorbance values were converted to nmol of residual AA using an external calibration curve. The OP activity was calculated as the slope of the linear regression of nmol of residual AA versus time.

DTT and AA solutions were freshly prepared. Samples were processed in sets of 10 and analyzed with both assays within 24 h of extraction to minimize the loss of redox-active species. Instrument calibration and blank measurements were performed every 10 samples. Field blanks were extracted and analyzed following the same procedure applied to PM samples; their mean depletion rate was subtracted from each sample value. Positive controls were included throughout the campaign: 9,10-phenanthrenequinone (PQ; CAS 130-15-4, Sigma Aldrich) for the DTT assay and dihydrate CuCl_2_ (VWR international) for the AA assay. Negative controls were performed using the ultrapure Milli-Q water (18 MΩ·cm) used for sample extraction. The uncertainties were estimated by replicated measurements on a subset of samples and are reported in the Supporting Information (Table S1). OP values were calculated, for the two assays, normalized in mass (subscript M) or in volume (subscript V).

### 2.4 Source Apportionment Approach

Source apportionment was done using the EPA PMF5 (positive matrix factorization) receptor model to identify and characterize the main natural and anthropogenic sources acting at the ECO and MAGA sites. PMF is one of the most widely used receptor models, able to operate with limited a priori information on sources.^34,35^ PMF was run on an input data set of 38 chemical species and 300 samples obtained pooling together the samples collected at the two sites to improve robustness of the results.^36,37^ This approach is justified considering that the chemical composition observed at the two sites is very similar (section 3.5), suggesting that the same set of sources is acting at the two sites eventually with different contributions. This is reasonable considering that the two sites are only less than 5 km one from the other, and it agrees with previous analysis in this area in which data from two or more nearby sites have been pooled together for PMF application.^14,38^ The NO_2_^–^ and the water-soluble organic nitrogen (WSON, discussed in detail in section 3.3) were classified as weak (tripled uncertainties) on the basis of both the signal-to-noise (*S*/*N*) criteria^39^ and the percentage of data above the detection limits.^36^ PMF was run using PM_2.5_ as the total variable, and the best solution identified 10 factors/sources. Some constraints were applied to the base solution to improve the separation between the identified sources arising from previous experience in this area and in other areas of south Italy.^13,14,24^ The focus is on a better separation of long-range dust from local soil and a better separation of biomass burning from traffic sources. These were the following: pull up maximally (K)_s_ and OC in biomass burning profile, where this is justified by the correlations of (K)_s_ with biomass burning tracers (levoglucosan, mannosan, and galactosan) showing Pearson correlation coefficients >0.83 at both sites; pull up maximally EC in traffic profile; pull up maximally (K)_i_, (Mg)_i_, and (Ca)_i_ in long-range dust profile. The analysis reported in section 3.4 clearly suggests (K)_i_, (Mg)_i_, and (Ca)_i_ as potential tracers of long-range dust so that they have been maximized in this profile. Previous work^13,14^ showed that, in this area, there could be an underestimation of OC in the biomass burning profile and an underestimation of EC in traffic. The combination of constraints used allowed us to limit these effects. The base run and constrained run profiles have been compared in Figure S9, showing limited changes in the PMF solution. Constrained runs were performed that enhanced the physical meaning of the chemical profiles without significantly affecting source apportionments. The final d*Q* change, compared to the base run, was 4.4%, which is an acceptable value considering that increases up to 8% are accepted in the current literature.^40^ The choices made in the PMF run as well as the results of diagnostic tests performed are reported in Supporting Information (Section S2).

The estimates of the contributions of the different sources to OP^DTT^_V_ and OP^AA^_V_ were done with three different methods based on two approaches. The first approach was based on the application of a multilinear regression (MLR) analysis between the daily contributions to PM_2.5_ found by the model PMF (independent variables) and the daily measured oxidative potential (either OP^DTT^_V_ or OP^AA^_V_ as dependent variables). The fitting β coefficients (i.e., the slopes resulting from MLR), representing the intrinsic contributions of each source to the oxidative potential were obtained using the XLSTAT tool with the intercept equal to zero. This assumes that PMF can resolve measured PM_2.5_ with negligible nonreconstructed values (as in this case see section 3.7) and it is a choice done in other studies.^17,24^ The MLR was evaluated using both ordinary least-squares fit (OLS) and weighted least-squares fit (WLS). Inverse variance WLS was applied, introducing a weighting term for individual OP observations whose variance is assumed to be related to the variance of the residuals.^41^ The second approach was to include OP^DTT^_V_ (or OP^AA^_V_) as total variable in the PMF input data set and perform two additional runs of PMF, one of each OP assay. Including OP in the input data set of PMF is an approach used in several different studies.^18,20,42–44^ In this approach the measured OP is put in direct association with the chemical composition rather than to a linear relationship with the contributions of sources. The latter approach allows to have constrained positive contributions of the different sources and to weight measured OP with its uncertainty.

## 3 Results and Discussion

Average concentrations of all quantified chemical species and of the oxidative potential are reported in Table S2 together with the interquartile range (between 25th and 75th percentiles). The seasonal and spatial variabilities of chemical compositions and source contributions were investigated for statistical significance by using the analysis of variance (ANOVA) test with a p-values threshold of 5%. In this work when it is indicated a statistically significant difference or trends it means that it was obtained *p* < 0.05 while when it is indicated a negligible or limited variability it means that it is not statistically significant, i.e., *p* ≥ 0.05.

### 3.1 Mass Closure and Charge Balance

Organic matter (OM) can be evaluated as OM = 1.6 OC where the factor 1.6 accounts for the non-C atoms in organic matter mass concentration.^45^ Without direct measurements of OM, the ratio OM/OC is affected by uncertainty so that it has been used the same ratio already applied to other studies in this area to maintain the comparability.^14,38^ Secondary inorganic aerosol (SIA) was evaluated as sum of NO_3_^–^, NH_4_^+^, and non-sea-salt sulfate (nss-SO_4_^2–^ = SO_4_^2–^ – 0.25Na).

The observed correlations between Na^+^, Cl^–^, and Mg^2+^ indicate a marine contribution due to sea-spray at both sites. The Cl/Na ratios were, on average, 0.50 (±0.05) at ECO site and 0.47 (±0.05) at MAGA site, significantly lower than the expected value (1.81) in seawater.^46^ This indicates a significant chloride depletion suggesting that sea-spray reaching the sites is relatively aged due to chemical reactions involving NaCl and nitric acid,^47^ observed also in previous studies in this area.^48^ Marine contribution can be evaluated assuming that all Na was from sea salt as 2.81Na, to limit the problem due to chloride depletion in the calculation.^14^

The crustal contribution was calculated as 1.15­(1.89Al + 2.14Si + 1.67Ti + 1.4­(Ca)_i_ + 1.2­(K)_i_ + 1.36Fe) that consider metal oxides of Al, Si and Fe, plus the water insoluble fraction of K and Ca.^24^ Carbonates were calculated from non-sea-salt calcium and magnesium as 1.5 nss-Ca^2+^ + 2.5 nss-Mg^2+^.^49^ The factor 1.15 takes into account sodium and magnesium oxides.^50^ The non-sea-salt component of Ca^2+^ was evaluated as nss-Ca^2+^ = Ca^2+^ – 0.038 Na^+^ and that of Mg^2+^ as nss-Mg^2+^ = Mg^2+^ – 0.129 Na^+^.

Results are reported in Figure S3. The chemical species detected but not used in the above-mentioned calculations were summed up and indicated as “other”. At both sites the measured species explain approximately 70% of measured PM_2.5_. This is a percentage comparable or slightly larger than those observed in other studies.^24,49^ The unexplained mass is mainly due to the water content, which could vary between 20% and 35%,^51^ and to the uncertainty on the coefficient used for the evaluation of OM.^45^ The differences statistically significant among the two sites were observed for EC, larger at the urban site MAGA because of the contribution of road traffic, and carbonates that are larger at the urban background site (ECO) likely because this is a local (i.e., short-range) contribution due to resuspension of dust. This is stronger at the ECO site because of the larger area of bare soil surrounding the site compared to the MAGA site and to the less frequent cleaning of the roads. The other observed contributions do not show statistically significant spatial variability.

The charge balance was investigated comparing the total charge (in neq/m^3^) of measured anions and cations in Figure S4. The closure is very good for both sites. The small observed anion deficit larger at ECO site, even if not statistically significant, could be due to nondetected carbonates because it is compatible and with the small contributions of carbonates shown in Figure S3: 2.0% (±0.15%) of PM_2.5_ at ECO site and 1.4% (±0.15%) at MAGA site.

### 3.2 Solubility of Major Chemical Species

The availability of measurements of total concentrations of Na, Cl, Mg, K, Ca, Br from ED-XRF with simultaneous determination of the corresponding water-soluble ions allowed determination of the soluble, indicated with ()_s_, and insoluble, indicated as ()_i_, fractions of these species. The difference OC – (OC)_s_ was used to determine the insoluble fraction of the organic carbon (OC)_i_. Br, Na, and Cl appeared to be almost completely soluble without differences statistically significant with between total concentrations and measured ions in agreement with observations of Kyotani and Iwatsuki.^52^ Comparison of soluble and total fractions of the other species is reported in Figure 1. The solubilities observed at the two sites were comparable within the standard errors. The observed solubility of OC was ranging from 62% (±2%) at ECO to 65% (±1.5%), in agreement with previous observations in this area,^14^ and comparable or slightly larger than the solubility observed in other regions such as eastern Spain (43%),^53^ Tianjin China (67%),^54^ and Gwangju, Korea (55% summer and 43% winter).^55^ Solubilities of the other elements ranged from 65% (Mg) up to 89% (Ca) and were comparable to literature results from Kyotani and Iwatsuki.^52^ The insoluble fractions (K)_i_, (Mg)_i_, and to a lower extent (Ca)_i_ were mainly associated with long-range transport of dust from Africa. During the two campaigns, it was observed 25 days influenced by long-range advection of dust. These events (in the following SD, i.e., Saharan Dust events or SD cases) were identified following the approach of Conte et al.^27^ based on concentrations of tracers of crustal material, mainly Si and Ti; on the daily forecast of African dust transport of the BSCDREAM8-CAMS model (https://dust.aemet.es/), on the 5-day back-trajectories calculated with HYSPLIT 4 arrival heights (200, 500, 1000, and 2000 m) at 2 h of the day (midday and midnight). Figure S5 shows the correlation of these insoluble fractions with the crustal contribution, evaluated as in section 3.1, separating SD and no-SD cases showing a very good correlation of (K)_i_, (Mg)_i_, and (Ca)_i_ with dust intrusion suggesting that these species could be considered good tracers of these events. (Ca)_i_ showed more scatter in the data, i.e., a lower correlation, likely because of the mixing with local calcium-rich soil.^56^

**Figure 1:**
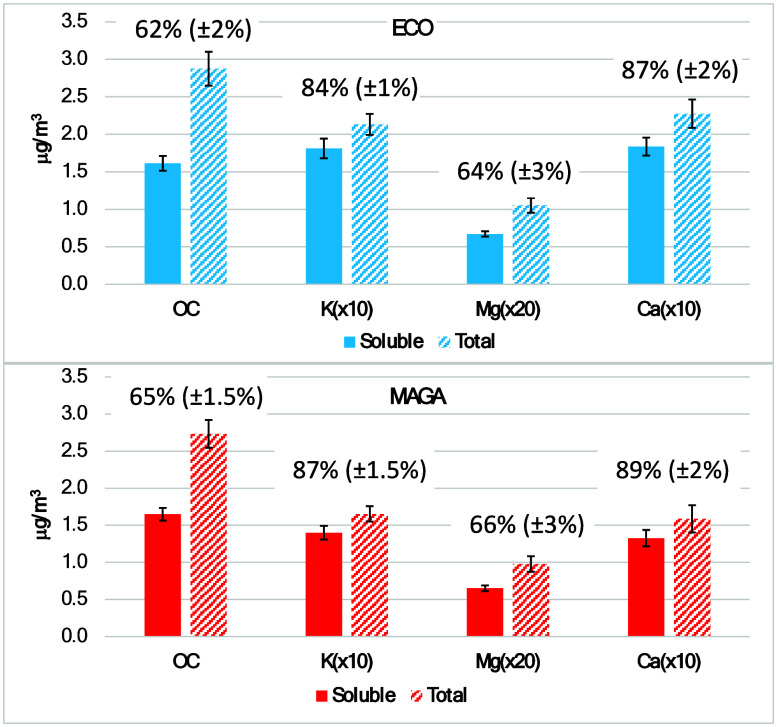
Soluble and total concentrations of OC, K, Mg, and Ca at ECO (a) and MAGA (b) sites. Percentages reported represent the average solubility of the different species. (×10) and (×20) indicate concentrations multiplied by these factors for a better readability. Differences between soluble and total fractions were statistically significant for all species reported in (a) and (b).

### 3.3 Determination of Water-Soluble Organic Nitrogen

The differences between WSTN measured by the TOC-TN analyzer and the total water-soluble inorganic nitrogen evaluated as the sum of nitrogen contained in measured ions WSIN = NO_2_^–^ + NO_3_^–^ + NH_4_^+^ were used to evaluate the water-soluble organic nitrogen WSON = WSTN – WSIN. This is a parameter that was found to have the strongest associations with levels of pro-inflammatory cytokines in lung epithelial cells (A549) compared to PM_2.5_ concentration and other chemical species.^57^ Figure 2 shows that WSON and WSIN concentrations at the two sites are comparable with WSON representing 15% ± 2% of total soluble nitrogen at ECO (13% winter and 16% summer) and 16% ± 2% at MAGA (14% winter and 17% summer). There are no measurements of WSON on PM_2.5_ available in Italy. However, it is possible to compare these numbers with the results of a two-week measurement campaign in north Italy, at the outskirts of the Po Valley, where an average of 23.7% was found in PM_10_.^58^ In two sites of southeastern USA an average of 9% was found^59^ while 23% was found in northern California.^60^ In Japan, it was found an average of 15.8%.^61^ Significantly larger values were found in continental China 44% ^62^ and in Hong Kong 28%.^63^ Differences in WSON concentrations across the different studies could be due a number of reasons such as differences in sources; photochemical aging; or differences in the measurement techniques. WSIN observed here is comparable with values found in other studies in the same area.^14^

**Figure 2:**
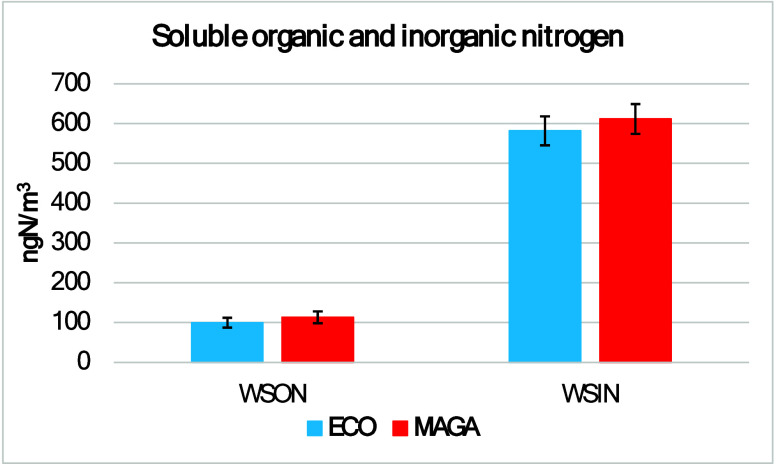
Comparison of the average soluble organic and inorganic nitrogen observed at the two sites.

### 3.4 Influence of African Dust on PM_2.5_ Composition

Average PM_2.5_ concentration during SD events were not statistically significantly different from the average concentration in absence of events (no-SD). At ECO average PM_2.5_ values were 14.8 (±0.7) μg/m^3^ (no-SD) and 15.5 (±1.3) μg/m^3^ (SD). At MAGA average PM_2.5_ values were 14.4 (±0.6) μg/m^3^ (no-SD) and 15.5 (±1.4) μg/m^3^ (SD). However, the increases of crustal contributions, evaluated as described in section 3.2, were 3.1 μg/m^3^ and 3.0 μg/m^3^ at ECO and MAGA, respectively. This suggests that the increase of crustal material during SD events is partially compensated by the decrease of the contribution of other sources. To investigate the change in the chemical composition of PM_2.5_, the normalized ratio

$${\mathrm{NR}}_{\mathrm{SD}}=\frac{{\left(x\right)}_{\mathrm{SD}}/{\left(x\right)}_{\mathrm{no‐SD}}}{{\left({\mathrm{PM}}_{2.5}\right)}_{\mathrm{SD}}/{\left({\mathrm{PM}}_{2.5}\right)}_{\mathrm{no‐SD}}}$$

for the average concentration of each chemical species *x* was investigated. Results are reported in Figure S6 only for the chemical species that had statistically significant differences when comparing SD and no-SD periods. All species related to biomass burning and to secondary inorganic aerosol were depleted during SD events. There was an obvious increase of the soil-related species (such as Mn, Fe, Al, (Ca)_s_, (Mg)_s_, Si, Ti, Sr) and an increase of Na and Cl suggesting a larger contribution of fresh seas spray during SD events that was hypothesized also in a previous study in this area.^14^ The increase of (K)_i_, (Mg)_i_, and (Ca)_i_ is comparable to the increase of Si and Al confirming that these species could trace long-range dust intrusion events. It was also observed the increase of some species Ni, P, Rb, and Cr generally considered of anthropogenic origin. This behavior was observed also in Tenerife (Spain) and interpreted as due to the transport pathways from regions affected by industrial emissions in Tunisia, Algeria, and Morocco.^64^ However, the increase of these elements is lower than the increase of Al and Si and they are present in the typical composition of African dust^65^ with a certain variability in abundances associated with the area of dust origin. In this data set, the increase of these elements is lower than the increase of Al and Si. Additional information is gained evaluating the crustal enrichment factors (EFs) comparing the concentration ratios of each specifical chemical species with a reference species (i.e., Al)^66^ in collected samples and in the average soil upper crust.^67^ This was done using the thresholds determined in Cesari et al.,^56^ that allowed a certain variability for local soil composition. Figure S7 shows the comparison of EFs for no-SD and SD cases at the two sites indicating that the EFs of all metals is lower during SD events. Even species having significant enrichment (EF ≥ 10) or mixed origin (5 < EF < 10) during no-SD cases had prevalent natural origin (EF < 5) during SD events. This suggests that, in this area, the values of NR_SD_ > 1 in Figure S6 are driven by the long-range dust contribution and by the sea spray transported with dust rather than by anthropogenic emissions.

### 3.5 Spatial Variability of Observed Concentrations

The average PM_2.5_ concentrations at the two sites are not statistically different (Table S2); however, there are significant differences in chemical composition arising from the different impacts of specific sources. Figure 3 compares the ratios MAGA/ECO of the average concentrations of the different chemical species. Only species having statistically significant difference are included in the figure, and the information is reported for the whole data set and separately for the winter and summer periods. Results show that EC and Cu, indicators of primary traffic emissions, were larger at the urban site; the same happened for succinate. The latter was identified as a species related to primary traffic emissions and to secondary aerosol formation from combustion-related emissions, especially during the summer period.^68,69^ The larger primary and secondary traffic-related contributions at the urban site appear to be compensated by the lower impact of other sources. (K)_s_, levoglucosan, mannosan, and galactosan, tracers of biomass burning emissions, showed lower concentrations at the urban site. The same happens for mannitol and glucose, interpreted as a lower impact of primary biogenic emissions (spores and fungi, discussed in more detail in section 3.7). The lower concentration of (Ca)_s_ at MAGA suggests a smaller contribution of local resuspended soil that is calcium-rich in this area of Italy.^56^

**Figure 3:**
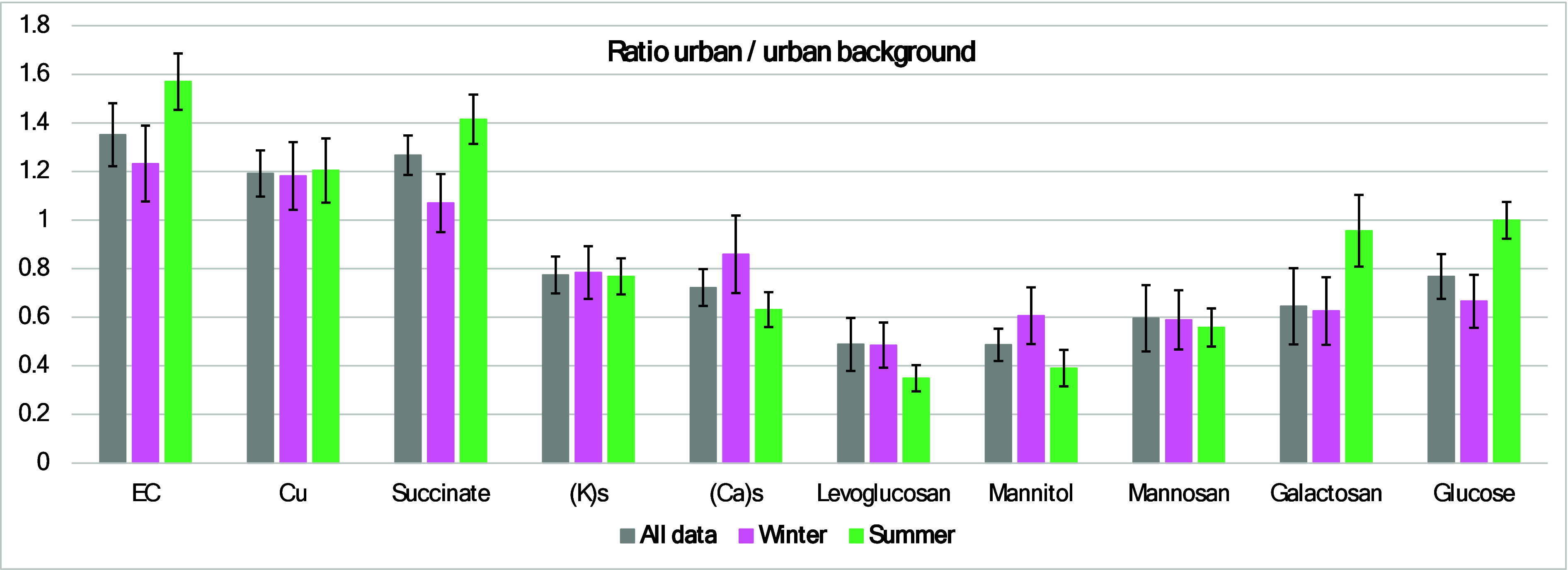
Ratio among concentrations observed at the two sites (MAGA/ECO) limited to chemical species that had differences statistically significant at the two sites. The ratios are reported for averaging all the data and separately for the winter and summer campaigns.

### 3.6 Seasonal Variability of Observed Concentrations

The average PM_2.5_ concentrations at the two sites are slightly lower during the summer campaign compared to the winter campaign. The summer reductions are approximately 11% at ECO and 9% at MAGA, but they are not statistically significant. To investigate seasonal variability, the normalized ratios

$${\mathrm{NR}}_{\mathrm{season}}=\frac{{\left(x\right)}_{\mathrm{winter}}/{\left(x\right)}_{\mathrm{summer}}}{{\left({\mathrm{PM}}_{2.5}\right)}_{\mathrm{winter}}/{\left({\mathrm{PM}}_{2.5}\right)}_{\mathrm{summer}}}$$

were evaluated for each species *x* and reported in Figure S8 only for the species that had statistically significant differences among the two seasons. Results show that there are competing seasonal trends. Combustion-related species from both traffic and biomass burning sources were larger during the winter period; the same apply for marine contribution likely due to a more favorable meteorology for advection from the sea. Instead, secondary inorganic (sulfate and ammonium) as well as secondary organic (WSON, succinate, oxalate) sources were larger during the summer period; with the exclusion of nitrate that has limited thermal stability so that it is larger during the winter as already observed at this site.^14^ These contrasting trends limits the seasonal variability of PM_2.5_ even if the relative changes of the sources are statistically significant.

### 3.7 Source Apportionment of PM_2.5_

The factors/sources chemical profiles, identified by the PMF5 are shown in Figure S9. The first factor was characterized mainly by mannitol and, to a lower extent, by glucose. This was interpreted as primary biogenic aerosol (PBA) from fungal spores or plant debris. A similar factor was observed in other studies with the same interpretation.^70,71^ In addition, Bauer et al.^72^ defined arabitol and mannitol as tracers for the quantification of airborne fungal spores. The second factor was mainly characterized by (Ca)_s_ and (Mg)_s_ and interpreted as local soil resuspended and possible contribution of construction works that were going on in proximity of both sites. The third factor was characterized mainly by EC, Cu, and Cr and was interpreted as due to primary traffic emissions. This profile is comparable to that found for traffic in a previous study in this area.^14^ Cu and Cr are elements abundant in several parts of vehicles and also associated with break and tire wear.^73^ The OC/EC ratio in this profile is approximately 0.8. This is in the range of the OC/EC ratios found in Europe for primary traffic emissions, ranging from 0.3 up to 2.9 with the lowest values mainly associated with diesel emissions and the largest values to gasoline fuels.^74^ Our ratio suggests the contribution of a mixed vehicle fleet including diesel and gasoline vehicles. The fourth factor was characterized by succinate, oxalate, and OC and it could be interpreted as secondary organic aerosol. Succinate was observed to be related to emissions and photo-oxidation of fossil fuel combustion.^68^ Oxalic acid dominated the PMF profile of SOA in PM_2.5_ collected in Beirut.^75^ The load of WSON support this interpretation. The fifth factor represents secondary nitrate. The sixth factor is characterized by Na (but not Cl) and (Mg)_s_ and it is interpreted as aged marine source. A similar profile was also found in France by Dominutti et al.^9^ The seventh factor was loaded with levoglucosan (L), mannosan (M), and galactosan (G) interpreted as biomass burning. The ratios L/M were 6.8 (±2) at ECO and 5.6 (±1.7) at MAGA while the ratios L/(M+G) were 4.7 (±1.5) at ECO and 3.7 (±1.2) at MAGA suggesting that softwood was the type of vegetation burned.^76^ The ratio L/(K)_s_ in this profile was approximately 1.3, comparable to the range observed in other studies for wood combustion Puxbaum et al.^77^ The eight factor was characterized mainly by sulfate and ammonium, and it was interpreted as secondary sulfate, however, it is also loaded with WSON suggesting a possible slight mixing with secondary organics. The nineth factor was loaded with Cl and Na with Cl/Na = 2, comparable with the average value in seawater (1.81), interpreted as fresh sea spray. The tenth factor was characterized by crustal elements Ti, Si, Al, and the insoluble fractions (K)_i_, (Mg)_i_, (Ca)_i_, and it was interpreted as long-range transport of dust. This profile is loaded with the tracers of Saharan dust advection that emerged in section 3.4, shown in Figures S5 and S6. It is quite common in this area to resolve two crustal sources one mainly associated with local resuspension (rich in Ca as the local soil of this area of Italy) and one mainly associated with metal oxides from long and medium range transport.^38^ What emerged from this analysis is that the detailed chemical data set used, including WSON and some organic sugars and acids, allowed to resolve ten sources compared to the usual seven^24^ or eight sources^14^ found in this area in previous studies relying on more traditional data sets. Specifically, it was possible to separate SOA and SIA and to resolve a component of primary biogenic aerosol. In addition, it was improved the separation of combustion sources (i.e., traffic and biomass burning) that was a source of uncertainty for source apportionment in this area.^14^

Figure S10 compares the measured and PMF-reconstructed PM_2.5_ at the two sites showing a very good agreement and that the receptor model was able to reconstruct concentrations without unexplained fraction. Figure 4 compares the average absolute contributions at the two sites, highlighting the contrasting source trends previously inferred from the chemical composition analysis (Ssection [Sec sec3.5]). Long-range dust, sea spray, aged marine aerosol, and sulfate show relatively uniform contributions, consistent with their regional-scale origins. In contrast, primary traffic emissions and secondary organic and nitrate aerosols (both influenced by gaseous traffic precursors) exhibit higher impacts at the urban site. Conversely, biomass burning, local soil dust, and fungal spores contribute more at the urban background site, reflecting the greater use of biomass for domestic heating in surrounding areas, the higher abundance of bare soils, and less frequent road cleaning.

**Figure 4:**
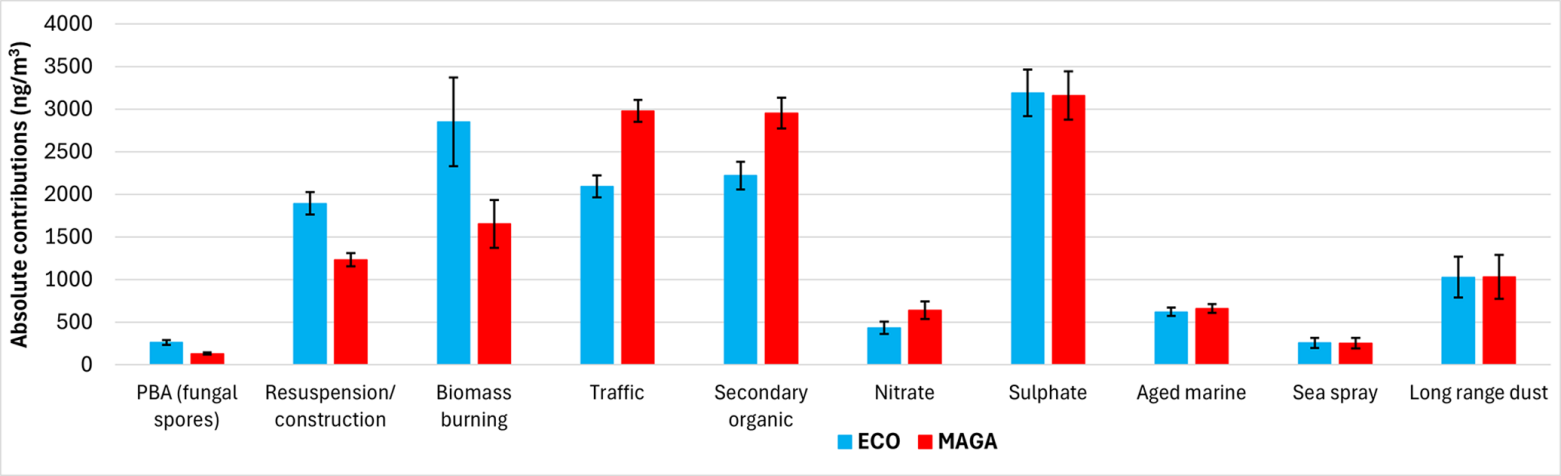
Absolute average contributions (and standard errors) of the different factors/sources identified by the PMF to PM_2.5_ at the two sites. Error bars represent the standard errors.

The seasonal trends of the identified sources, expressed as the ratio of their contributions during the summer and winter campaigns, are illustrated in Figure S11. These trends, consistent across both sites, are driven by variations in the primary emissions and meteorological conditions. Local soil exhibits a higher contribution during the summer, likely due to drier soil conditions facilitating resuspension. Similarly, secondary organic aerosol and secondary sulfate increase in summer due to intense regional photochemistry. In contrast, nitrate shows the opposite trend due to its thermal instability. However, the overall trend of SIA is primarily driven by sulfate, resulting in an impact approximately twice as large during the summer. Long-range dust, sea spray, and aged marine sources show higher contributions in the winter. Nevertheless, this pattern may stem from the frequency or intensity of specific events during the sampling period; thus, a multiyear data set is required to confirm its interannual stability. Biomass burning is negligible in summer, confirming its origin in domestic heating during the winter months; a similar seasonal pattern is observed for primary traffic emissions. The presence of these opposing seasonal trends dampens the overall seasonal variability at the two sites, despite the significant fluctuations of individual sources. These findings, even if limited to a specific area, are promising for suggesting season-specific air-quality mitigation strategies.

### 3.8 Source Apportionment of Oxidative Potential

Figure S12 illustrates the average OP at the two sites, separately for the summer and winter periods and normalized by both volume and mass. The results reveal divergent seasonal trends for OP^DTT^_V_ and OP^AA^_V_. OP^DTT^_V_ is higher during winter, particularly at the urban background site, likely due to the significant impact of biomass burning, that is a relevant contributor as shown in Figure 5 and Table S3. In contrast, OP^AA^ values are comparable across both sites, with higher levels observed during the summer. These discrepancies suggest that distinct emission sources drive the OP response evaluated by the two assays. To further investigate these relationships, a hierarchical cluster analysis (HCA) was performed using the Ward’s method based on Euclidean distance.^78^ The dendrograms for both sites, presented in Figure S13, show several source-specific clusters that are consistent with the sources identified by the PMF analysis. Levoglucosan, mannosan, and galactosan form a cluster associated with biomass burning, linked at a greater distance to OC, EC, and (K)_s_. Additionally, a Na–Cl cluster reflects sea spray influence, while another cluster comprises various groups including crustal elements and insoluble fractions such as (Ca)_i_, (Mg)_i_, and (K)_ioxidati_ due to both dust advection and local soil. A third cluster consists of secondary inorganic aerosols, linked at a larger distance to secondary organic components due to their comparable seasonal trends (section 3.7). Notably, OP^DTT^_V_ and OP^AA^_V_ belong to different clusters. OP^DTT^_V_ is primarily linked to combustion sources (biomass burning tracers, OC, and EC) with some influence from metals such as Zn and Pb, particularly at the ECO site. Conversely, OP^AA^_V_ is mainly associated with metals (primarily Cu) and biological markers like mannitol and glucose, suggesting a possible influence of the identified PBA source on OP^AA^ levels. It has to be mentioned that OP^AA^_V_ is sensitive to soluble metals, but the solubility of metals, generally source-dependent, is not investigated in this study leading to some possible uncertainties in the interpretation of the drivers of OP^AA^_V_.

**Figure 5:**
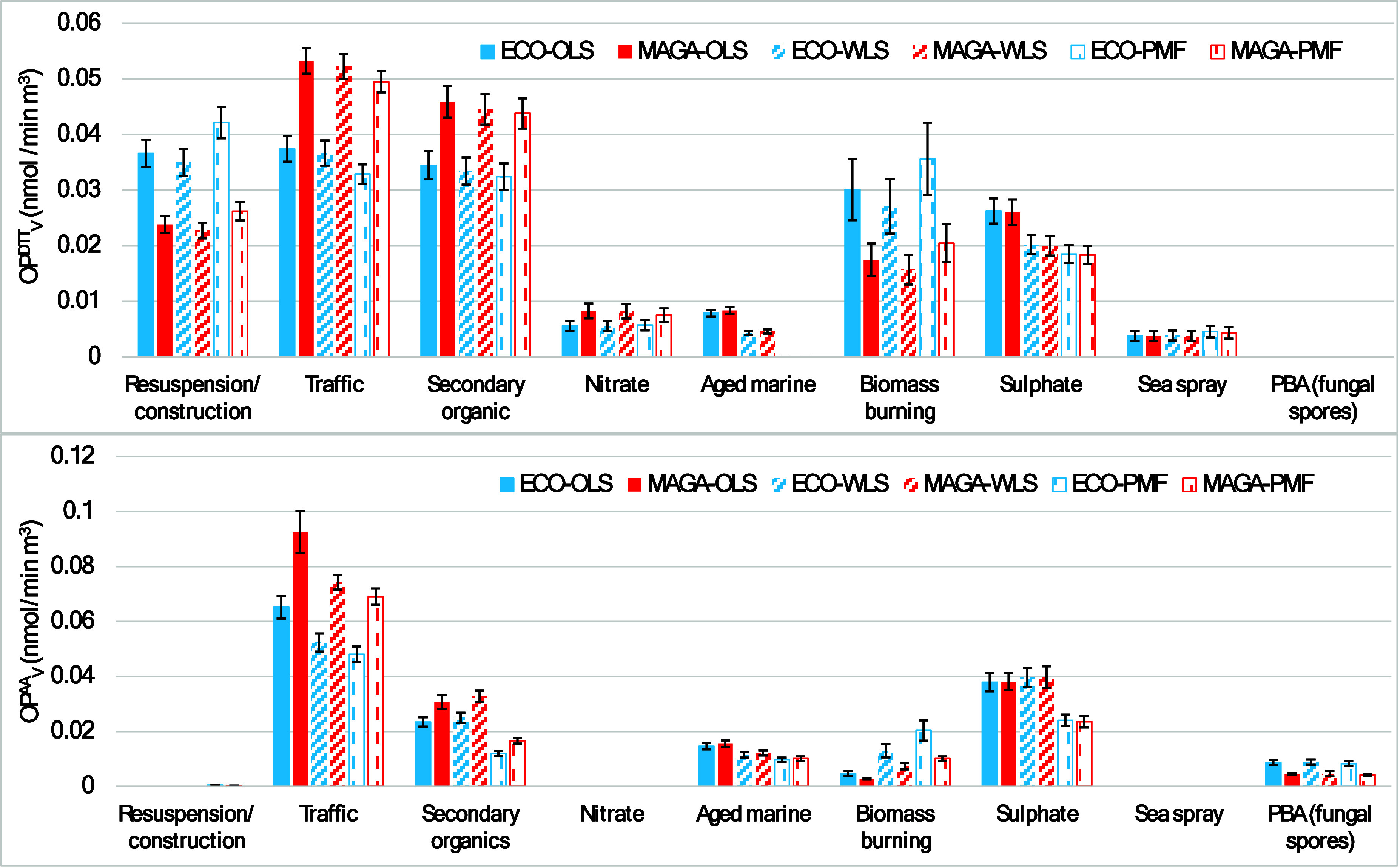
Comparison of the absolute average contributions (and standard errors) of the different sources, obtained with the three methods, to OP^DTT^_V_ (top) and OP^AA^_V_ (bottom) at the two sites.

Figure S13 illustrates the comparison between measured OP and the values reconstructed via source apportionment using three distinct approaches: PMF coupled with MLR (OLS), PMF coupled with MLR (WLS), and PMF alone. The PMF profiles with added OP were characterized by the same sets of tracers as the initial ones used for apportionment of PM_2.5_. The Pearson distance among the profiles with and without OP was lower than 0.05 for all profiles at both sites, suggesting an extremely good comparability of the profiles. The regression coefficients for the MLR applications are detailed in Table S3, together with the intrinsic coefficients evaluated with the PMF-only approach. The source apportionment models demonstrated a higher reconstruction efficiency for the OP^DTT^ assay compared with OP^AA^. Specifically, the unexplained fraction of OP^DTT^ was 3% using the OLS approach and 10% for both the WLS and PMF-alone methods. In contrast, the unexplained variance for OP^AA^ was notably higher: 10% for the OLS, 15% for the WLS, and 31% for the PMF alone. OP^AA^ is mainly driven by trace metals, and this discrepancy suggests that OP^AA^ is primarily driven by specific trace metals that may not be fully captured by sources identified through PMF. Furthermore, the available data set may not adequately account for the variability in the water solubility and oxidation state of trace metals across different emission sources observed in other studies.^79,80^ In addition, there could be synergic or antagonistic interactions between metals that are not captured by the source approaches used.

Source contributions to OP^DTT^_V_ and OP^AA^_V_ obtained at the two sites using the three different methods are shown in Figure 5, including only sources with statistically significant contributions. The three methods yielded consistent trends, although some discrepancies were observed for specific sources. Notably, OLS tended to overestimate contributions from aged marine aerosol (for both assays), sulfate (limited to OP^DTT^_V_), and traffic (limited to OP^AA^_V_) compared to WLS and PMF. The comparison of the three apportionment methods for OP should be tested also at other sites, however, the differences in this work are limited even if WLS may be a preferable approach for PMF-MLR applications as it accounts for measurement uncertainty and limits the effect of heteroscedasticity.

The relevant impact of biomass burning to OP^DTT^_V_ is comparable with observations in other studies in this area^13^ and in other European sites.^16^ In some different geographical contexts, biomass burning was identified as the main contributor to OP^AA^_V_ (e.g., Weber et al.^12^ and Dominutti et al.^9^). However, in multisite studies in other regions, it is shown that this aspect is site-dependent and that in some sites, like in those investigated in this work, traffic emissions are the major contributor to OP^AA^_V_.^81^ Secondary inorganic aerosol is expected to have a low redox activity from a chemical point of view. However, the PMF profiles are not those of pure substances and keep trace of the chemical signature of the main anthropogenic sources of the sulfate and nitrate precursors so that it is usually found a certain contribution to both OP^AA^_V_ and OP^DTT^_V_.^9,14,20^ Low but detectable contributions to OP of sea spray and aged marine sources were observed also in other studies,^9,13,81^ usually smaller compared to the contributions of combustion sources, as in these results. Relevant contributions of road dust to OP^DTT^_V_ of PM_2.5_ and, to a lower extent, to OP^AA^_V_ were also observed in in ’t Veld et al.^82^ Results consistently show, for both assays, a low intrinsic contribution of long-range dust to OP, consistent with the observations of Chirizzi et al.^83^

Spatial variability between the two sites was driven by the same sources influencing PM_2.5_ concentrations. For OP^DTT^_V_, higher contributions were observed for traffic, secondary organic aerosol, and nitrate, while lower contributions were attributed to biomass burning and resuspension/construction at the MAGA site. This consistency is expected given the strong correlation between OP^DTT^_V_ and PM_2.5_ (Pearson *R* ≥ 0.70, *p* < 0.05 at both sites). Although the correlation between OP^AA^_V_ and PM_2.5_ was significantly lower (*R* = 0.19 at ECO and *R* = 0.29 at MAGA, *p* < 0.05), the variability of source contributions followed similar drivers, except for resuspension/construction, which did not influence OP^AA^_V_.

Source drivers for OP^DTT^_V_ and OP^AA^_V_ exhibited distinct patterns. Sea spray, nitrate, and resuspension/construction contributed negligibly to OP^AA^ but significantly influenced OP^DTT^_V_, whereas primary biological aerosols (PBA, such as fungal spores) impacted OP^AA^ but not OP^DTT^_V_. While biomass burning and SOA affected OP^AA^_V_ less than OP^DTT^_V_, traffic emissions emerged as the primary driver of OP^AA^_V_ at both sites. Conversely, OP^DTT^_V_ was influenced by a broader range of sources, including traffic, SOA, resuspension/construction, and biomass burning, the latter particularly at the ECO site. During the winter, traffic and biomass burning were the dominant sources, accounting for approximately 50% of OP^DTT^_V_ and 60% of OP^AA^_V_. In summer, the contributions of sulfate and SOA became more prominent, especially for OP^DTT^. These varying source weights underpinned the divergent seasonal trends: OP^AA^_V_ peaked during the summer, while OP^DTT^_V_ was either comparable across seasons (at MAGA) or higher during winter (at ECO). Assuming OP is an effective indicator of adverse health effects, these findings are promising for the implementation of season-specific mitigation strategies, such as targeting biomass burning during the cold period and resuspension/construction emissions in the warm period, the latter potentially via intensified road cleaning and dust suppression at construction sites.

Figure S15 compares the relative contributions of natural and anthropogenic sources to OP^DTT^_V_, OP^AA^_V_, and PM_2.5_ mass, showing PMF-only analysis. Results consistently show that traffic contributes more to both OP assays relative to its contribution to total mass, whereas the opposite trend is observed for sulfate. Cumulative secondary aerosols (inorganic and organic) exert a significantly greater impact on OP^DTT^_V_ than on OP^AA^_V_. Additionally, while SOA contributions to OP^DTT^_V_ scale with mass at the urban background site, a disproportionately higher contribution to OP was observed at the urban site, likely due to the higher oxidative potential of traffic-induced SOA.

## Supplementary Material



## Notes

Additional details of MDL; concentrations and uncertainties of chemical species; diagnostic of PMF; impact of sources (DOCX)
