# Supplementary material for: Unraveling the Different Drivers of PM2.5 Mass and Oxidative Potential at Two Sites of Southern Italy
Source: Environ Sci Technol. 2026 May 25;60(22):16044–56. doi: 10.1021/acs.est.6c02676 (PMC13262043; doi:10.1021/acs.est.6c02676)
Supplement: Supplementary file 1 [file es6c02676_si_001.docx]

**Unravelling the different drivers of PM_2.5_ mass and oxidative potential
at two sites of southern Italy**

Serena Potì^1, 2^, Laura Martina^1, 3^, Florin Unga^1^, Daniela Cesari^1^, Adelaide Dinoi^1^,
Antonio Pennetta^1^, Ermelinda Bloise^1^, Paola Semeraro^1^, Giuseppe Deluca^1^, Luca Cirillo Ciricugno^1^,
Livia Giotta^3^, Maria Giulia Lionetto^3^, Lucio Calcagnile^4^, Annarosa Mangone^5^,
Maria Rachele Guascito^1, 3, *^, Daniele Contini^1, *^

^1^ Istituto di Scienze dell’Atmosfera e del Clima (ISAC), Consiglio Nazionale delle Ricerche (CNR), Str. Prv. Lecce-Monteroni km 1.2, Lecce, 73100, Italy

^2^ Dipartimento di Ingegneria dell’Innovazione, Università del Salento, Via Per Monteroni 165, Lecce, 73100, Italy

^3^ Dipartimento di Scienze e Tecnologie Biologiche ed Ambientali DiSTeBA, Università del Salento, Via Per Monteroni 165, Lecce, 73100 Italy

^4^ Dipartimento di Matematica e Fisica, Università del Salento, Via Per Arnesano, Lecce, 73100 Italy

^5^ Dipartimento di Chimica, Università degli Studi di Bari, Via Orabona 4, Bari, 70121, Italy

* Corresponding authors

E-mail: [daniele.contini@cnr.it](mailto:daniele.contini@cnr.it)

E-mail: [maria.rachele.guascito@unisalento.it](mailto:maria.rachele.guascito@unisalento.it)

**Pages = 20**

**Number of figures = 15**

**Number of tables = 3**

**Section S1 - Uncertainties of measured chemical species**

The concentration of a specific species and sample was quantified if it was larger than the standard deviation σ_B_ of the blank filters; otherwise, a threshold value equal to σ_B_/2 was considered. In cases in which the concentration was below the MDL, or not detectable above the average variability of the field blanks, a concentration value equal to the maximum between the MDL/2 and σ_B_/2 was assumed. The uncertainty for concentrations < MDL or not distinguishable from blanks were assumed to be 100%, whereas the uncertainty for quantified concentration, *C_ij_*, of the sample i relative to the species j, was calculated as in Cesari et al. (2018), using the equation: $\Delta C_{i,j}=\frac{\sigma_{B,j}}{2}+h_{j}C_{i,j}$. The parameters and the MDLs are reported in Table S1.

| **Species** | **MDL (ng/m^3^)** | **σ_B_/2 (ng/m^3^)** | **h** |
| --- | --- | --- | --- |
| PM_2.5_ | 600 | 300 | 0.02 |
| EC | 10 | 10 | 0.05 |
| OC | 10 | 10 | 0.05 |
| (OC)_s_ | 30 | 15 | 0.05 |
| (OC)_i_ | - | 15 | 0.05 |
| WSTN | 50 | 25 | 0.05 |
| WSON | - | 10 | 0.10 |
| Na | 40 | 40 | 0.05 |
| Mg | 2.2 | 1.0 | 0.05 |
| Al | 0.3 | 0.1 | 0.10 |
| Si | 1.5 | 0.7 | 0.05 |
| P | 0.05 | 0.2 | 0.10 |
| SO_4_^2-^ | 28 | 10 | 0.05 |
| Cl | 0.5 | 5.0 | 0.05 |
| K | 1.0 | 1.0 | 0.05 |
| Ca | 0.27 | 3.0 | 0.05 |
| Ti | 0.08 | 0.02 | 0.05 |
| Cr | 0.07 | 0.02 | 0.05 |
| Mn | 1.0 | 0.4 | 0.10 |
| Fe | 3.0 | 0.9 | 0.10 |
| Ni | 0.1 | 0.05 | 0.10 |
| Cu | 1.5 | 0.4 | 0.10 |
| Zn | 0.16 | 1.5 | 0.10 |
| Br^-^ | 0.19 | 0.05 | 0.05 |
| Rb | 0.27 | 0.1 | 0.15 |
| Sr | 0.25 | 0.05 | 0.15 |
| Pb | 0.3 | 0.22 | 0.15 |
| NO_2_^-^ | 6.0 | 3.0 | 0.15 |
| NO_3_^-^ | 9.0 | 5.0 | 0.05 |
| C_2_O_4_^2-^ | 3.0 | 1.0 | 0.05 |
| Succinate | 0.8 | 1.0 | 0.05 |
| NH_4_^+^ | 1.8 | 2.0 | 0.05 |
| (K)_s_ | 1.8 | 1.0 | 0.05 |
| (K)_i_ | - | 1.5 | 0.10 |
| (Mg)_s_ | 1.3 | 1.0 | 0.05 |
| (Mg)_i_ | - | 1.0 | 0.10 |
| (Ca)_s_ | 0.9 | 3.0 | 0.05 |
| (Ca)_i_ | - | 2.6 | 0.10 |
| Levoglucosan | 0.06 | 0.07 | 0.05 |
| Mannitol | 0.09 | 0.05 | 0.05 |
| Mannosan | 0.34 | 0.21 | 0.05 |
| Galactosan | 0.17 | 0.23 | 0.05 |
| Glucose | 0.08 | 0.13 | 0.05 |
| Mannose | 0.07 | 0.05 | 0.05 |
| OP^DTT^_V_ | 0.002 | 0.002 | 0.10 |
| OP^AA^_V_ | 0.002 | 0.002 | 0.10 |

Table S1) Parameters used for the evaluation of uncertainties of the dataset. OP is reported nmol/min m^3^. OC, (OC)_s_, (OC)_i_, and EC are in ngC/m^3^ while WSTN and WSON, are in ngN/m^3^.

**Table S2**

|  | **ECO** | **MAGA** |
| --- | --- | --- |
| PM_2.5_ | 14844 (10500 - 17400) | 14556 (10400 - 16600) |
| OC | 2876 (1335 - 3174) | 2733 (1461 - 2877) |
| EC | 536 (235 - 581) | 724 (426 - 83) |
| (OC)_s_ | 1614 (821 - 1958) | 1649 (1005 - 1858) |
| (OC)_i_ | 1264 (330 - 1462) | 1086 (348 - 1251) |
| Na | 199.5 (73.6 - 243.8) | 195.9 (77.8 - 244.2) |
| Mg | 52.7 (19.4 - 61.8) | 49.1 (17.8 - 53.4) |
| Al | 83 (17.9 - 77.5) | 80.5 (18 - 73.7) |
| Si | 234.9 (58.4 - 217.2) | 228.1 (58.6 - 207.6) |
| P | 2.17 (1.57 - 2.34) | 2.22 (1.58 - 2.47) |
| SO_4_^2-^ | 2008 (1047 - 2765) | 1942 (990 - 2638) |
| Cl | 98.8 (5.7 - 57.8) | 92.1 (5.4 - 57.9) |
| K | 211.9 (111.1 - 233.3) | 165.2 (79.9 - 201.6) |
| Ca | 227.5 (91.8 - 285.5) | 158.6 (77.8 - 158.2) |
| Ti | 7.73 (0.16 - 7.12) | 7.53 (0.02 - 6.65) |
| Cr | 0.26 (0.14 - 0.30) | 0.30 (0.20 - 0.33) |
| Mn | 2.47 (1.16 - 2.85) | 2.13 (1.06 - 2.49) |
| Fe | 72.45 (31.25 - 81.53) | 73.5 (37.34 - 72.95) |
| Ni | 0.097 (0.043 - 0.044) | 0.118 (0.043 - 0.044) |
| Cu | 1.81 (0.92 - 2.30) | 2.15 (1.34 - 2.74) |
| Zn | 8.92 (3.99 - 11.46) | 8.16 (3.97 - 10.41) |
| Br^-^ | 2.50 (1.98 – 3.00) | 2.36 (1.75 - 2.94) |
| Rb | 0.37 (0.09 - 0.47) | 0.39 (0.11 - 0.54) |
| Sr | 1.10 (0.04 - 1.17) | 1.04 (0.04 - 1.17) |
| Pb | 2.73 (1.35 - 3.21) | 2.25 (1.13 - 2.52) |
| NO_2_^-^ | 6.34 (0.70 - 7.92) | 5.73 (0.69 - 4.50) |
| NO_3_^-^ | 436.3 (134.4 - 500.8) | 514.0 (117.8 - 609.3) |
| C_2_O_4_^2-^ | 159.9 (96.0 - 213.2) | 152.6 (85.9 - 204.9) |
| Succinate | 29.3 (15.9 - 41.1) | 37.2 (19.4 - 54.3) |
| NH_4_^+^ | 618.8 (175.2 - 1016) | 635.2 (226.4 - 998.9) |
| (K)_s_ | 180.6 (85.6 - 201.9) | 139.9 (66.9 - 159.6) |
| (K)_i_ | 31.2 (1.5 - 36.5) | 25.4 (1.5 - 26.6) |
| (Mg)_s_ | 33.6 (17.5 – 42.0) | 32.7 (15.9 - 40.5) |
| (Mg)_i_ | 19.1 (1.0 - 17.3) | 16.4 (1.0 - 12.3) |
| (Ca)_s_ | 183.6 (79.5 - 233.1) | 132.6 (68.3 - 145.2) |
| (Ca)_i_ | 43.9 (1.0 - 42.3) | 26.0 (1.0 - 21.5) |
| WSON | 99.7 (10.0 - 145.8) | 113.1 (11.0 - 121) |
| WSIN | 582 (228 - 829) | 611 (266. - 831) |
| WSTN | 659 (257 - 918) | 715 (287 - 979) |
| Levoglucosan | 113.32 (3.83 - 101.96) | 55.34 (1.23 - 57.91) |
| Mannitol | 1.38 (0.17 - 1.93) | 0.67 (0.18 - 0.91) |
| Mannosan | 16.6 (0.73 - 12.22) | 9.89 (0.48 - 9.42) |
| Galactosan | 7.49 (0.24 - 4.47) | 4.84 (0.26 - 4.13) |
| Glucose | 7.08 (3.12 - 8.46) | 5.44 (2.82 - 6.24) |
| Mannose | 2.72 (0.55 - 2.23) | 2.25 (0.52 - 2.14) |
| OP^DTT^_V_ | 0.200 (0.127 - 0.241) | 0.177 (0.122 - 0.210) |
| OP^AA^_V_ | 0.190 (0.099 - 0.231) | 0.187 (0.103 - 0.231) |
| OP^DTT^_M_ | 0.0140 (0.0104 - 0.0169) | 0.0126 (0.0104 - 0.0150) |
| OP^AA^_M_ | 0.0149 (0.0077 - 0.0150) | 0.0139 (0.0078 - 0.0163) |

Table S2) Average concentrations of the different chemical species and of OP at the two sites. Inter-quartile range (IQR, between 25^th^ and 75^th^ percentiles) in parentheses). Data is in ng/m^3^ for all species while OP is reported normalised in volume (nmol/min m^3^) and in mass (mnol/min µg). OC, (OC)_s_, (OC)_i_, and EC are reported in ngC/m^3^ while WSTN, WSON, and WSIN are reported in ngN/m^3^.

| **Source** | **OP^DTT^**  **MLR with OLS**  **β (nmol/min µg)** | **OP^DTT^**  **MLR with WLS**  **β (nmol/min µg)** | **OP^DTT^**  **PMF only**  **β (nmol/min µg)** | **OP^AA^**  **MLR with OLS**  **β (nmol/min µg)** | **OP^AA^**  **MLR with WLS**  **β (nmol/min µg)** | **OP^AA^**  **PMF only**  **β (nmol/min µg)** |
| --- | --- | --- | --- | --- | --- | --- |
| Resuspension/construction | 0.0193  (0.0136-0.0251) | 0.0185  (0.0141-0.0229) | 0.0218  (0.0197-0.0240) | - | - | 0.0002  (0.0002-0.0003) |
| Traffic | 0.0179  (0.01 – 0.0248) | 0.0175  (0.012 – 0.0232) | 0.0162  (0.0152-0.0173) | 0.0311  (0.0213-0.0409) | 0.0249  (0.0215-0.0284) | 0.0231  (0.0214-0.0248) |
| Secondary organic | 0.0155  (0.0112-0.0199) | 0.0151  (0.0114-0.0187) | 0.0147  (0.0133-0.0161) | 0.0104  (0.0038-0.0170) | 0.0111  (0.0085-0.0137) | 0.0055  (0.0049-0.0061) |
| Nitrate | 0.0129  (0.0036-0.0223) | 0.0129  (0.0013-0.0243) | 0.0123  (0.0095-0.0152) | - | - | - |
| Aged marine | 0.0127  (0-0.0253) | 0.0069  (0-0.0156) | - | 0.0235  (0.0067-0.0402) | 0.0183  (0.0132-0.0235) | 0.0155  (0.0138-0.0172) |
| Biomass burning | 0.0106  (0.008-0.0131) | 0.0095  (0.007-0.0120) | 0.0125  (0.0092-0.0157) | 0.0016  (-0.0021-0.0053) | 0.0044  (0.0028-0.0061) | 0.0071  (0.0052-0.0090) |
| Sulphate | 0.0082  (0.0058-0.0107) | 0.0063  (0.0043-0.0083) | 0.0058  (0.0051-0.0065) | 0.0120  (0.0082-0.0158) | 0.0125  (0.0108-0.0143) | 0.0075  (0.0066-0.0084) |
| Sea spray | 0.0148  (0.0028-0.0268) | 0.0151  (0.0069-0.0232) | 0.0175  (0.0117-0.0233) | - | - | - |
| PBA (fungal spores) | - | - | - | 0.0344  (-0.0133-0.0822) | 0.0352  (0.0178-0.0527) | 0.0318  (0.0266-0.0369) |
| Long range dust | - | - | - | - | - | - |
| Not reconstructed fraction | 3% | 10% | 10% | 10% | 15% | 31% |

Table S3) Results obtained from multi-linear regression (MLR) analysis of measured OP^DTT^_V_ and OP^AA^_V_ and contributions of the different sources, found by the PMF receptor model, for the two sites. Results are reported using both OLS and WLS, only sources giving statistically significant contributions to OP are reported.

**Section S2 – Details on the PMF run**

Source apportionment was done using the EPA PMF5 (Positive Matrix Factorization) receptor model run on an input dataset of 38 chemical species and 300 samples. Previous studies in this area allowed to characterise eight sources of PM_2.5_ using an input dataset with a lower chemical detail (Cesari et al., 2018). Therefore, PMF model was run starting from seven up to 11 factors.

The best solution for the base run was obtained using ten factors/sources. The determination of the optimal number of factors with a reasonable physical meaning was achieved analysing the parameters IM (the maximum individual column mean), and IS (the maximum individual column standard deviation), obtained from the scaled residual matrix, together with Q-values (goodness of fit parameter). When the number of factors increased to a critical value, IM and IS parameters experienced a marked change (Cesari et al., 2016). The ten factors found (Q_robust_ 53319.8) have a reasonable physical interpretation and the analysis of the scaled residual were centred on zero and symmetrically distributed for almost all variables, with the vast majority of the values in the range ±3, meaning that the model was able to reasonably fit the different chemical species.

Displacement was used to evaluate influence of rotational ambiguity. Application of displacement showed no swaps for every dQ level. The results for the different factors are showed in Figure S1 that shows generally narrow confidence intervals for most species, especially for the key tracers of each factor, indicating stable and well-defined factor profiles.

Successively, the constraints discussed in the main text were applied to the base solution to improve the separation between the identified sources with a final dQ change, compared to the base run, of 4.4%, that is an acceptable value considering that increases up to 8% are accepted in current literature (Crova et al., 2024). The chemical profiles of constrained and base run were analogous, characterised by the same major tracers, showing limited differences for the key tracers of each factor. They are compared in Fig. S9.

The bootstrap methodology (Paatero et al., 2014) was applied to evaluate uncertainties in PMF results. The bootstrap of the constrained solutions was evaluated with 500 runs using random seed and R=0.6 as threshold. Results showed a limited number of swaps (less than 7% for all factors) between long-range dust profile with resuspension/construction work and nitrate profile. Uncertainties evaluated with bootstrap are included in the comparison of profiles (Fig. S9). This confirms the robustness of the solution found.

**Figure S1**

**Resuspension/construction**


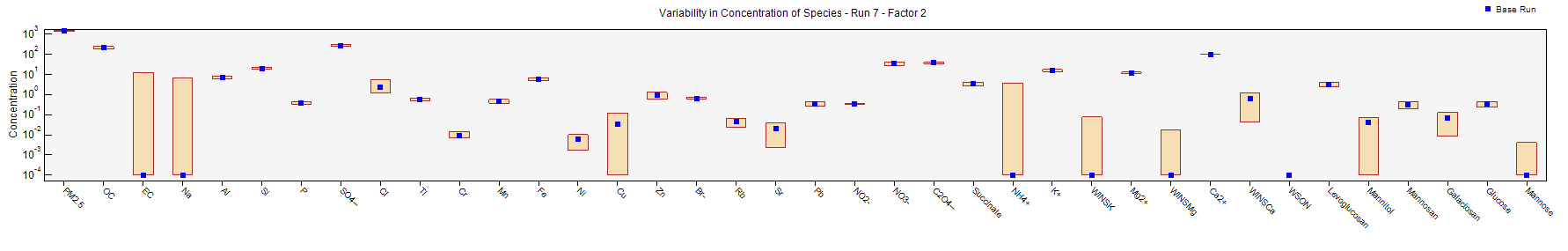

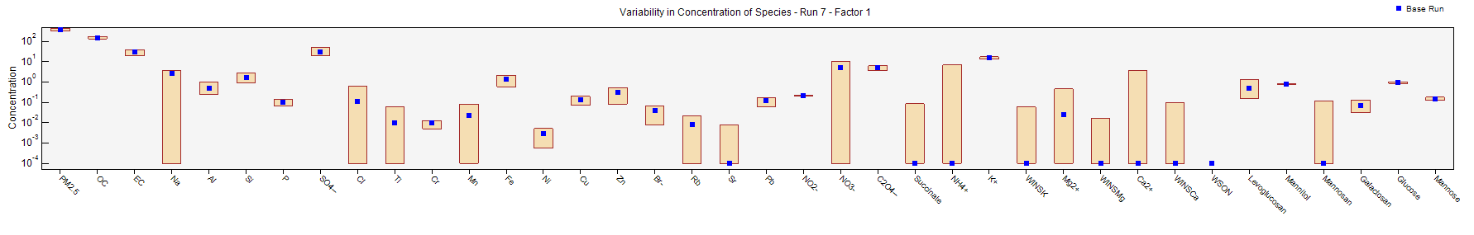


**PBA (fungal spores)**

**Traffic**


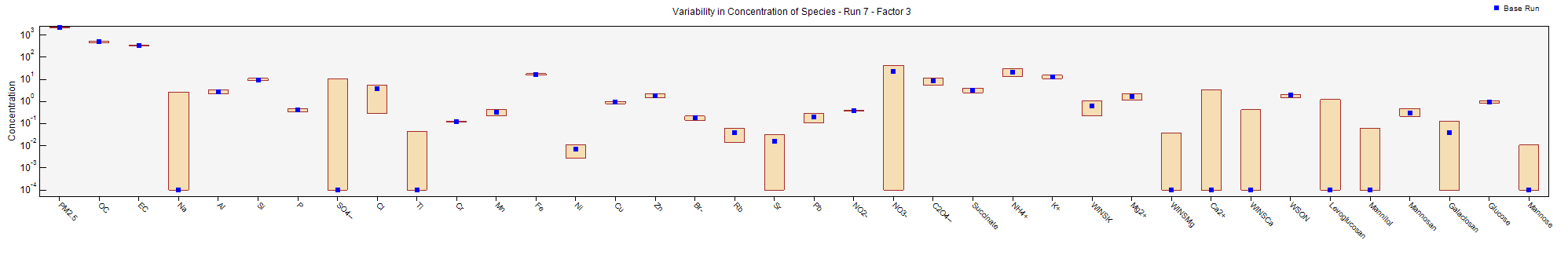


**Secondary organic**


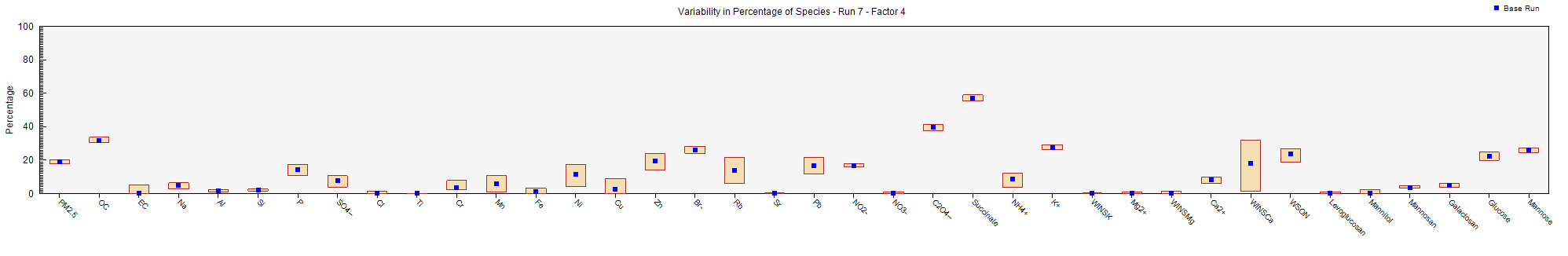


**Nitrate**


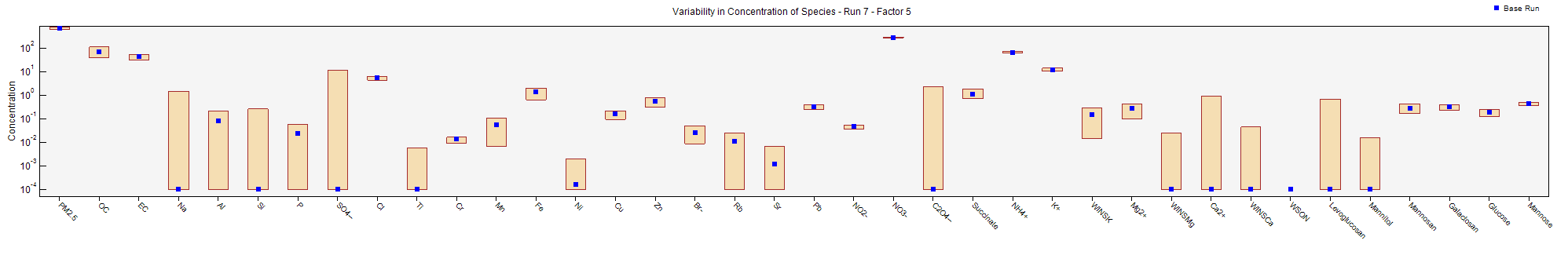


**Aged marine**


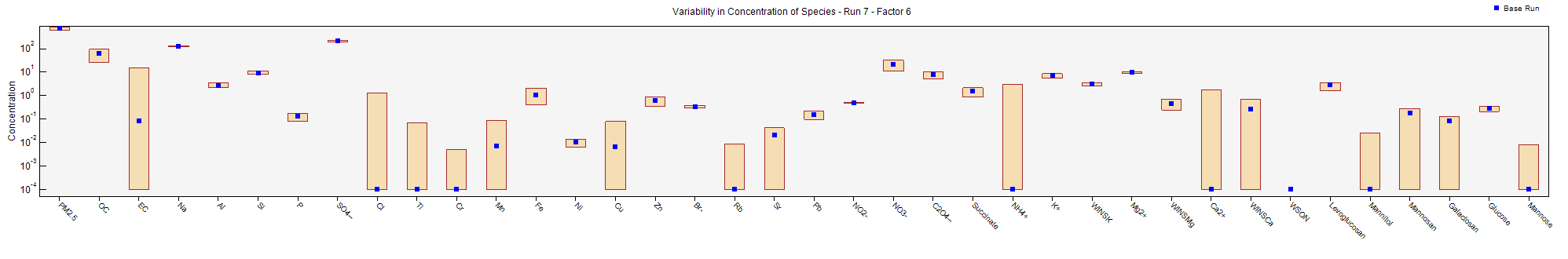


**Biomass burning**


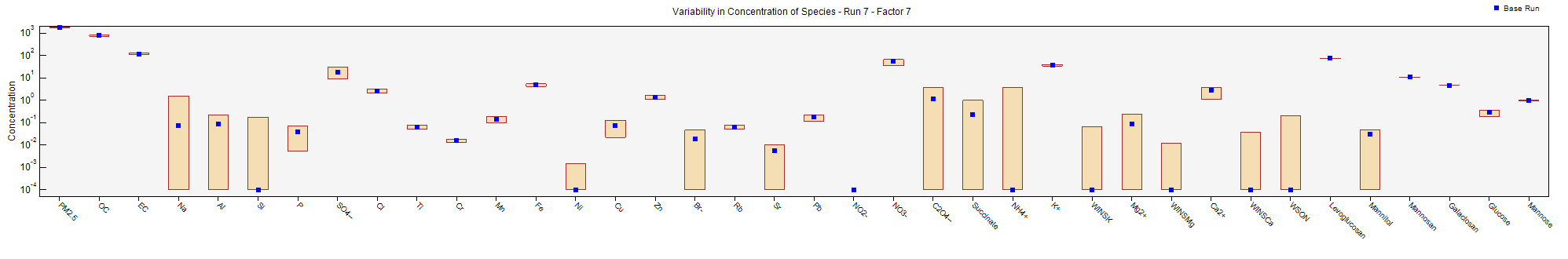


**Sulphate**


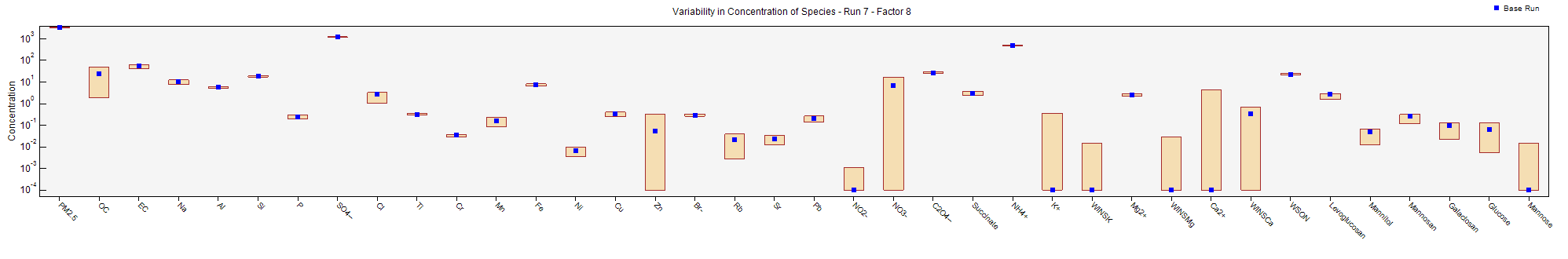


**Sea spray**


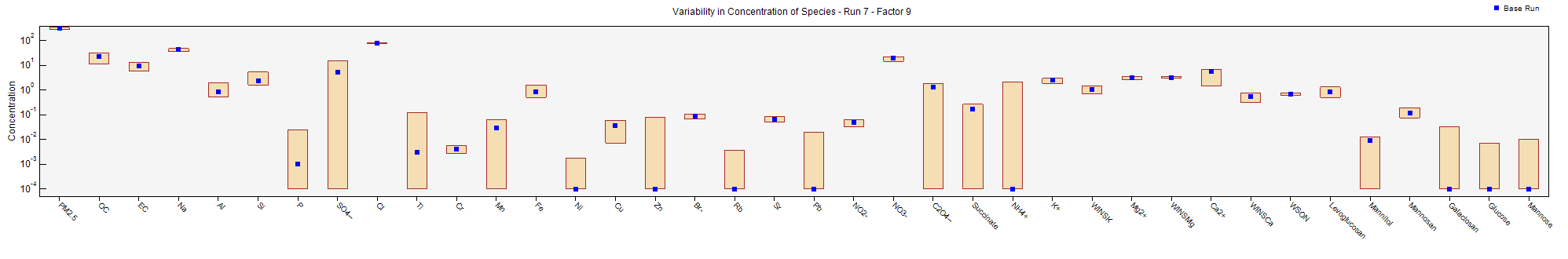


**Long range dust**


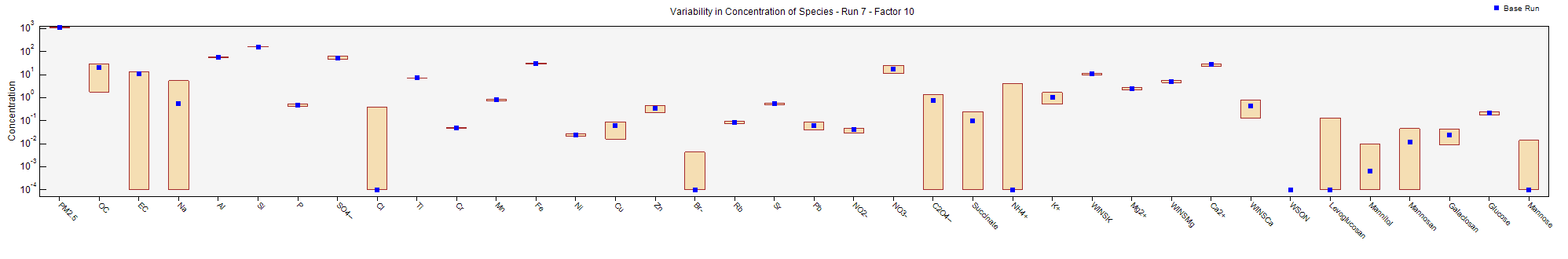


Fig. S1) Illustration of the displacement results for the different PMF profiles.

**Figure S2**

**
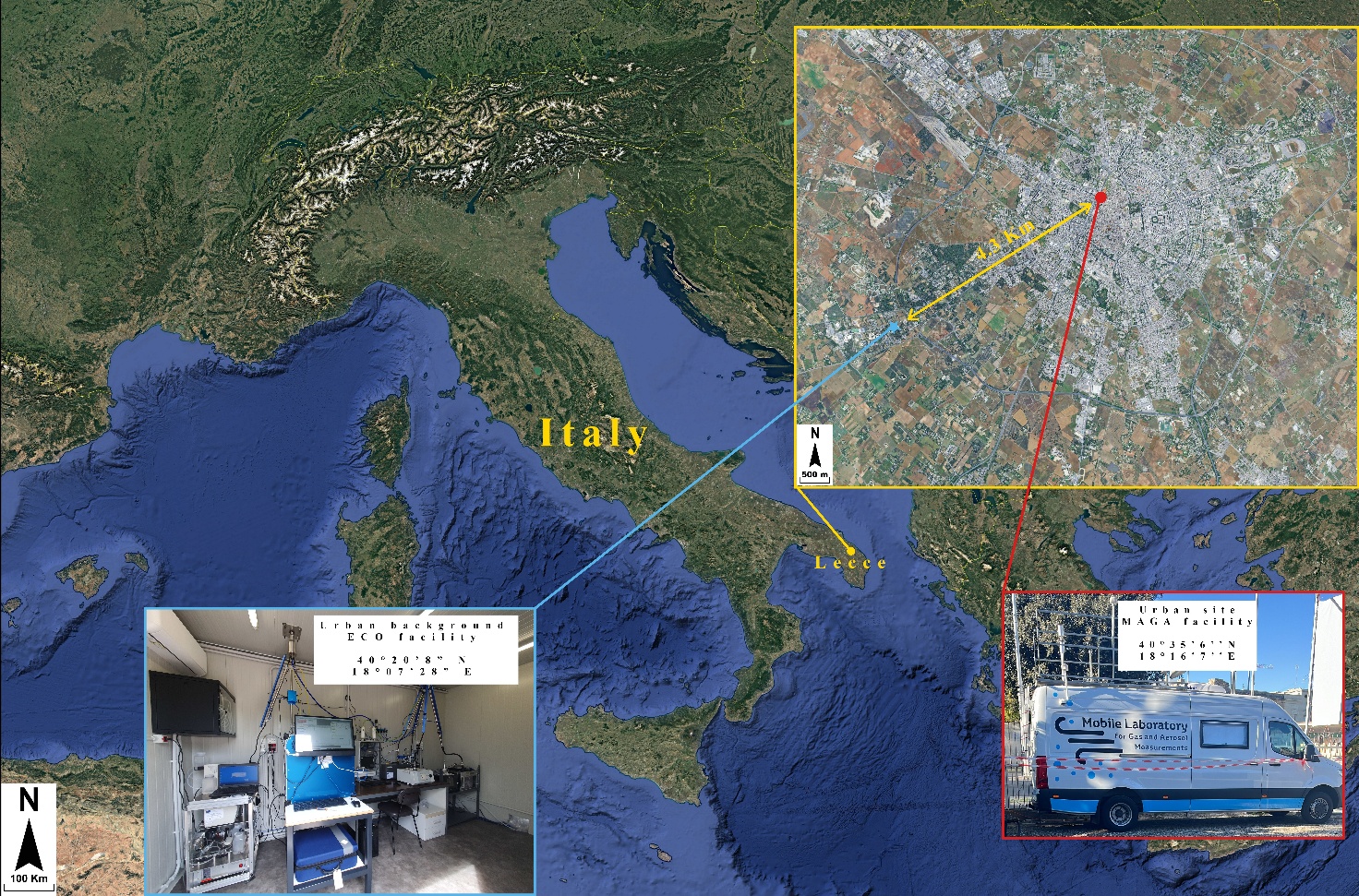
**

Fig. S2) Illustration of the positions of the two measurements sites and of the facilities used.

**Figure S3**

*)

*)

Fig. S3) Results of mass closure at the two sites (top) and absolute contributions (bottom) of the chemical species and sources evaluated through stoichiometric analysis. Graph includes also indication of statistically significant differences (*) and percentages of PM_2.5_. SIA and OM were divided by 5 and indicated as (/5).

**Figure S4**

Fig. S4) Charge balance at the two sites (top) and comparison of the average sum of charges in anions and cations (bottom).

**Figure S5**

Fig. S5) Scatter plot showing the correlation between crustal contributions to PM_2.5_ and insoluble fractions (K)_i_, (Mg)_i_, and (Ca)_i_ at the two sites. The events of intense dust transport from Africa have been highlighted in red.

**Figure S6**

Fig. S6) Normalised ratios of concentration observed during SD events and in absence of events for the two measurement sites. Only species presenting statistically significant differences between SD and no-SD are reported.

**Figure S7**

Fig. S7) Enrichment factors separating events of dust advection from non-event days. Significant enrichment suggesting anthropogenic origin (EF≥S2); mixed origin (S1<EF<S2); mainly crustal origin (EF<S1) according to Cesari et al. (2012).

**Figure S8**

Fig. S8) Normalised ratios of concentration observed during winter and summer campaigns. Only species presenting statistically significant differences between the two seasons are reported. The values were divided by 10 for the species indicated with “/10”.

**Figure S9**

Fig. S9) PMF Profiles with concentration of species (bars) and percentages (marks). Constrained run (filled bars and marks), base run (empty bars and marks). Errors obtained with bootstrap.

**Figure S10**

Fig. S10) Comparison of PM_2.5_ reconstructed by the PMF with the measured values.

**Figure S11**

Fig. S11) Ratio of the summer to winter contributions of sources to PM_2.5_ at the two sites.

**Figure S12**

Fig. S12) Average values of OP^DTT^ and OP^AA^, normalised in mass and volume, at the two sites separated per season. Data is reported normalised in volume and in mass.

**Figure S13**

Fig. S13) Results of the cluster analysis separated for the two sites

**Figure S14**

Fig. S14) Comparison of the OP^DTT^_V_ and OP^AA^_V_ values reconstructed by the source apportionment with the measured values. Results are reported separated per site and per source apportionment approach. Green points (two for DTT and four for AA measurements) were excluded, because out of the linear trend for more than tree standard deviations, from the fits but not from the analysis of the data.

**Figure S15**

Fig. S15) Relative contributions of the different sources to OP^DTT^_V_, OP^AA^_V_, and PM_2.5_ evaluated with PMF-only method at the two sites. Only sources giving statistically significant contributions to at least of the assay are reported.

**References**

Cesari D, De Benedetto GE, Bonasoni P, Busetto M, Dinoi A, Merico E, Chirizzi D, Cristofanelli P, Donateo A, Grasso FM, Marioni A, Pennetta A, Contini D., 2018. Seasonal variability of PM_2.5_ and PM_10_ composition and sources in an urban background site in Southern Italy. Sci Total Environ 612, 202–213.

Crova, F., Bernardoni, V., Cadeo, L., Canepari, S., Hopke, P.K., Massimi, L., Perrino, C., Valli, G., Vecchi, R., 2024. Multi-time and multi-size resolution receptor modeling to exploit jointly atmospheric aerosol data measured at different time resolutions and in multiple size classes. Atmos. Environ. 333, 120672.

Paatero, P., Eberly, S., Brown, S.G., Norris, G.A., 2014. Methods for estimating uncertainty in factor analytic solutions. Atmos. Meas. Tech. 7(3), 781–797.
